# Supplementary material for: Postnatal depression and its social-cultural influences among adolescent mothers: A cross sectional study
Source: PLOS Glob Public Health. 2023 Jun 23;3(6):e0002025. doi: 10.1371/journal.pgph.0002025 (PMC10289363; doi:10.1371/journal.pgph.0002025)
Supplement: Attachment — Submitted filename: Response to Reviewers 12.4.23.docx [file pgph.0002025.s001.docx]

The Editor

PLOSE Global Health

14/4/2023.

Dear Sir / Madam,

I would like to present our revised copy of manuscript titled **“postnatal depression and its social - cultural influences among adolescent mothers: A cross sectional study in Malawi”.** We have made the revisions to reflect what the reviewers suggested and where necessary we have provided some explanations. The responses are presented in a table. The first part responds to the first reviewer and the second part for the second reviewer.

We confirm that all authors have approved the manuscript for submission and the content of the manuscript has not been published or submitted for publication elsewhere.

I hope you will consider this manuscript for publication in your journal.

The responses

**Thank you for reviewing this manuscript. The comments are informative and relevant, we have tried to consider the suggestions from all the reviewers. These have been summarized below.**

| Reviewers comment | Responses |
| --- | --- |
| Abstract: 1. The information provided under background is exact copy of things written under the first three lines of the background in the main document of the manuscript. It should not be like this. | The information on the background has been changed |
| 2. The reason (S) why the author (S) intended to conduct this study was not clearly stated in the background section | This has been added in the abstract from line 14 – line 24 and more information added |
| 3. A cross-sectional survey was administered from September 2021 to March 2022 (Date should be specified) | The dates have been inserted as from 7^th^ September 2021 to 31^st^ March 2022 |
| 4. Very important components of the methods like sample size, sampling technique, data entry methods… were missed. | We have included the information as suggested |
| 5. On the conclusion you stated that, the prevalence of postnatal depression among adolescent mothers in Malawi is high. What was your reference or cut off point to describe as high or low? | the cutoff point has been added it is ≥10 as probable PND |
| Methods: 1. The information regarding defining the adolescent age range is not needed under study design and setting. It should better be included under operational definition or measurement. | This has been removed |
| 2. The source and study populations were not clearly specified. | We have Included information on source as points where mothers were recruited thus postnatal checkup clinic, under five clinics and family planning clinics.  The study population and inclusion criteria are further described in the section ‘study population and sampling’ – commencing line 145. A description of the region is described in the ‘study design and setting’ section – commencing page 128. |
| 3. Criteria for inclusion was written as age greater than 20 under the population, are these ages group your subject of interest? | Thank you. This was a typographical error. We have amended this to ≤19. Thank you for noting this |
| 4. One focus group discussion (FGD) with 15 adolescent mothers was conducted to determine the face validity of the survey. How can we use FGD to validate quantitative survey tool? | We have conducted face validity to determine the suitability of the survey for the target group. Face validity is a type of content validity that indicates the instrument is understandable by the target group. It was deemed important for this study that adolescents understood the language and questions. This is often done as a FGD (Liamputtong, 2013).  We have edited this section:  *Face validity of the survey was achieved via expert view in addition to a focus group discussion (FGD) with 15 adolescent mothers. Language and comprehension of questions were explored during the FGD. (Liamputtong, 2013)* |
| 5. How was the tool developed? Adapted or adopted? What was the alpha value for internal consistency? | This an adapted tool that was already used in Malawi among perinatal women. The alpha for internal consistency is indicated under measures /line 240 to 243 |
| 6. Data analysis was not well written | Thank you for your comments. We have re-written the Data analysis section (see line 239) |
| **Ethical statement**: lacks the following information • Authors reporting experiments on humans must confirm that all methods were carried out in accordance with relevant guidelines and regulations. |  |
| • Must include a sentence confirming that informed consent was obtained from all subjects and/or their from all subjects and/or their legal guardian(s).  Here as the study also involves illiterate population, please ensure to provide an additional statement confirming that informed consent was obtained from their respective LARs as well. | Thank you for this comment. We had addressed consent in the ‘procedure for data collection’ section. We have moved these sentences to the ethics section. We have added reference to the legal guardian (see lines 271-279)  We had also indicated that the project was approved by recognized ethic committees, however, have added an additional sentence:  *The research is governed by the principles of beneficence, justice and respect and in accordance with the ethical principles of the Declaration of Helsinki(Tembo et al., 2022).*  We have moved the statements around section to the ethics section and added a comment regarding the |
| Results 1. The calculated sample size was 395, but you invited 401 participants to participate in your study. Why do so? And your response rate became 99.5%. as to me you can’t invite more than the calculated sample size and your response rate should be 100% if you included 395 postnatal mothers. Try to differentiate nonresponse rate, excluded participants and incomplete data/information. | Thank you for your comment. The sample size we required (as outlined in the study population and sampling section) was 395. We continued to invite participants until we reached the required sample. Hence 401 participants were approached and agreed to read (or be read) the information about the project. Usually, the response rate would be calculated based on all who were approached divided by those who did participate.  We have added the words eligible adolescent mothers to the first sentence as adolescent mothers were first asked the eligibility criteria before being provided information. WE have reworded this sentence:  *Four hundred and one eligible adolescent mothers agreed to consider participation in this study*. |
| 2. Why not you include the first paragraph of the result section under subtitle sociodemographic chxs | Thank you. This has been amended |
| 3. The sum of the frequencies for some variables, such as polygamous marriage and marital age, does not equal 395. | Polygamous marriage: the calculation was only based on those that responded yes to marriage. Which is 308. Equally the age at marriage the denominator is 330. This excludes participants who have never been married |
| 4. According to Table 1, none of the participants have smoked or used tobacco in the last 12 months. As the other category is zero, it would be preferable to provide this information in text rather than a table. | I have removed the information. However, in future it can be a very good reference point considering the changes in social attitudes. |
| 5. The author (s) attempted to present factors linked with postnatal depression individually using chi square and logistic regression tables. It is preferable to combine both tables because the third table does not provide information on the strength and direction of the association. | Thank you for the feedback, however we thought that we should maintain the logistics table for readers to follow and avoid overcrowding the table3 However, to address your suggestion, we have added a column to give information on direction of association on the binary logistic table by adding the coefficient column to show the correlation |
| 6. What is the reason for the confidence interval being wide for the variables like IPV? | The reasons are possibly the following.  Convenience sampling which might have caused sampling error such that there was low variability in responses. We had included a sentence highlighting this potential limitation in the limitations section of the original manuscript (see Line 541).  Secondly could be that the responses that are biased due to social desirability bias. For instance, this study was happening in area where there is a campaign to reduce gender-based violence and fighting early marriages. In addition, adolescents were escorted to the clinics with their partners and therefore, they feared that the partners would be harshly punished and tend to provide not to tell the true responses. Adolescents were very sensitive when responding to IPV questions.  We have added the following to the limitations section:  *Given the sensitive nature of some of the questions, social desirability bias may have impacted some findings. (Bryman, 2016)* |
| 7. What parameters were considered for performing multivariable logistic regression analysis? Bivariate vs Multivariate analysis? confidence interval? COR? | Binary logistic regression. The outcome was binary no PND EPDS scores ≤ 10 and PND EPDS scores ≥10. This has been added to the data analysis section. |
| **Discussion** 1. What is your cut off point to say higher than, lower than and similar (inline)? | This has been included as above |
| 2. The authors justified the lack of studies on postnatal depression in Malawi, although in the discussion, it is said that the findings of this study are greater than the findings of two studies among postnatal adult women in Malawi in similar rural settings. This is perplexing! | Thank you for your comment. To our knowledge there are no studies focusing on PND among adolescent mothers in Malawi. We have made a comparison to PND for the adult population, as this is the only data available. |
| **Limitation** 1. What do you think about the reliability and validity of Edinburgh Postpartum Depression Scale (EPDS)? | Have added information on line 505 t0 508. This is about EPDS being a screening tool and not giving diagnosis.  We have discussed the validity of the EPDS in Malawi (see Line 215). In addition, we have added the following to the limitations section:  *It is recognised the EPDS is a screening tool and is not diagnostic however it is widely* |
| Reviewer 2 |  |
| **Abstract**  Very good overall. Based on the study findings, consider adding more information in the background section and the conclusion.  Consider revising your aim—something related to factors associated with postnatal depression among adolescent mothers. Since you have different variables, you are analyzing, consider expanding the aims from just prevalence. | Thank you for the comment, I have revised the abstract |
| **Introduction**   1. The background section has lots of meaningful information, but the sentences should be switched around for better flow /logic. For example, either start with the global prevalence, then funnel down to Malawi, or start with the description of postnatal depression, then move on to the epidemiology of the issue. Also   The aim is well explained in the introduction | Thank you for the observation. I have tried to introduce the adolescent pregnancy as an issue then linking with its risks as regards to postnatal depression and why postnatal is an issue among adolescent mothers |
| **Methods**  **Study Design and Setting**  Revise sentence flow. Line 110-111 is placed between two sentences describing the study site, which is somewhat confusing | Thank you for this. This has been amended |
| **Data collection Tool and measures**  What was the rationale for including perceived social support in the demographic section? | This is a social factor, so I have included it under social factor. Thank you for this observation |
| **Data Analysis**   1. What sort of bivariate analysis was conducted? Logistic regressions or linear regressions? | Binary logistic regression was used. We have edited this section and added more detail around the data analysis. |
| 2. Did you conduct any multivariable regression? since the study results in the abstract section report adjusted odds ratios. 3. This section should state the proposed analytical methods for | Thank you for your comment. We have reviewed the data analysis section |
| 4. each study - the dependent variables, dependent variables, and the covariates, if any, for each analysis conducted. | Thank you for your comment. We have reviewed the data analysis section and have included reference to dependent and independent variables. |
| **Results**  *Table 1*  Are the extra spaces intentional? | Thank you for your comment. We have edited the table. |
| Perceived social support is not reported in the results section. Was this an error? | This was an error we have reported as a score in Table one |
| ***Table 2 results in text***  The information is excellent and relevant, but it is not reader friendly. It would help if the findings reported in Table 2 were grouped. For example, group interactions with health care providers, religious leaders, and traditional healers together, then reactions from parents/ relatives together | I have grouped them as suggested |
| ***Table 3 results***  What is the meaning of ^a^ in Table 3? | ^a^ means a test result from the formular used by x^2^ and t test |
| ***Table 4***  Did you conduct multivariate or multivariable logistic regressions? If multivariate, what were the other dependent variables | Binary logistic regression. The data analysis section has been updated to reflect this. |
| In general, the results section should reflect the survey information reported in the methods section and be organized systematically.   - For example, the methods started with 1) demographic information and social factors, 2) behavioral factors; 3) The Edinburgh Postnatal Depression Scale (EPDS); and 4) questions about common cultural factors. | Thank you for your suggestion. We strived to reduce the word count. The survey has lots of information, so we have excluded some of the tables and just summarized the results of EPDS after tabulation into two categories to minimize the tables |
| - The results from the social behavioral section and the EPDS (ten-item scale) were not reported yet they were included in the methods. State the reason why only the overall IPV or social scores are reported. | Some variables (perceived social support, EPDS and IPV) were reported as scales. Individual items were not reported in an attempt to ensure the paper is concise. It is anticipated these items will be reported in more detail in subsequent papers. |
| Discussion   - Lines 328-330- Although other studies reported certain factors associated with postnatal depression among adolescent mothers, these findings were not explicit from your results. You have the data on educational level, support etc., so link your findings with other research. - Lines 352-360- Very interesting findings based on results. | We have given an explanation for the level of education. line 425 to 427  *Level of education was not significantly associated with depression in this study. This is because in this sample, approximately 99% of participants had not attended secondary education. This has made the responses to be clustered on primary school and there was lack of variability leading to education being not significant*.  And support line 399 – line 343  The study found a negative correlation *between MPSS and depression where by a decrease in MPSS support resulted in increase in depression, this is similar to many studies conducted among adult perinatal women ) An individual's ability to handle pressures is thought to be significantly improved by social support( Hence, adolescent mothers may have* benefited from extra support |
| - Lines 395-406: This section is good but could be made stronger. The authors should consider separating being prayerful and engaging in communal religious activities. Sometimes, individuals can engage in solitary prayer, which could be isolating but still reduces postnatal depression. Or individuals can be active in religious activities without necessarily being” very prayerful” and still have better outcomes. - Ensure consistency with terms throughout the paper. For | A very true observation. However, I have reviewed the section and rerun the logistics regression. The results point to being prayerful as correlating positively with depression. |
| - example, the abstract and discussion mention interaction with “health workers”, but the results mention interaction with “healthcare.” The two terms are not synonymous. | This is noted and changed as suggested |
| Overall, this is a well-written paper. However, the methods section (analysis) could be revised to reflect the results. As it is, the results are confusing because the reader is not aware of the analysis conducted. The discussion section is good. The author did well in comparing their findings with other studies. However, the paper could be made stronger by also including more implications on either policy or healthcare practices that can be incorporated to screen/ reduce adolescent postnatal depression. | Thank you for the review and your comprehensive and useful feedback |

Bryman, A. (2016). *Social research methods*. Oxford university press.

Tembo, C. P., Burns, S., & Portsmouth, L. (2022, May 13). Maternal mental health of adolescent mothers: a cross-sectional mixed-method study protocol to determine cultural and social factors and mental health needs in Lilongwe, Malawi. *BMJ Open, 12*(5), e056765. <https://doi.org/10.1136/bmjopen-2021-056765>

Sincerely yours,

Mrs Chimwemwe Tembo
