# Supplementary material for: Postnatal depression and its social-cultural influences among adolescent mothers: A cross sectional study
Source: PLOS Glob Public Health. 2023 Jun 23;3(6):e0002025. doi: 10.1371/journal.pgph.0002025 (PMC10289363; doi:10.1371/journal.pgph.0002025)
Supplement: Attachment — Submitted filename: Response to Reviewers 11.5.23.docx [file pgph.0002025.s002.docx]

The Editor

PLOSE Global Health

12/5/2023.

Dear Sir / Madam,

I would like to present our revised copy of manuscript titled **“postnatal depression and its social - cultural influences among adolescent mothers: A cross sectional study in Malawi”.**  We have made the revisions to reflect what the reviewers suggested and where necessary we have provided some explanations. The responses are presented in a table. We confirm that all authors have approved the manuscript for submission and the content of the manuscript has not been published or submitted for publication elsewhere. We have removed one reference Kawiya et al, because this article was not published, and Rice PL was incomplete and did not have a title.

The supporting file is Data, but we are still using this data and therefore, it should not be published now. This was attached for reviewing purposes.

I hope you will consider this manuscript for publication in your journal.

The responses

**Thank you for reviewing this manuscript. The comments are informative and relevant, we have tried to consider the suggestions. These are summarized below.**

| Reviewer 2 comment | responses |
| --- | --- |
| However, you still need to do some work on the methods and analysis section. The abstract reports aOR which are adjusted odds ratios, yet the study does not report any multivariable analysis. If only bivariate analyses were done, state the rationale for not conducting multivariable analyses and how you would account for confounding variables. | Binary logistic regression was used to model the relationship between a binary outcome variable and the predictor variables. In this study, we used binary logistic regression because the outcome variable is binary or dichotomous (probable Depression and No depression). It modelled the outcome based on the given predictors that I have outlined in the data analysis section as suggested; therefore, the data analysis section, has been updated to reflect this and in the abstract section I have included the sentence. Lines 26 and 32  The approach was to include the predictor variables as covariatea in the model. By including these variables in the model, the effect of the predictor variables on the outcome can be estimated while controlling for the potential influence of the confounding variables. We have listed the covariates from lines 238 to 246. The possible confounder was age. Some possible confounders, such as past psychiatric history, HIV serostatus, were not included in the model because they were not significant in the bivariate analysis. However, we did not account for physical health problems, and hormonal changes. This is acknowledged in the limitation section, lines 510 -512. |

Sincerely yours,

Mrs Chimwemwe Tembo
